# Efficiency of primary spine care as compared to conventional primary care: a retrospective observational study at an Academic Medical Center

**DOI:** 10.1186/s12998-022-00411-x

**Published:** 2022-01-06

**Authors:** Serena Bezdjian, James M. Whedon, Robb Russell, Justin M. Goehl, Louis A. Kazal

**Affiliations:** 1 Southern California University of Health Sciences, Whittier, CA 90604 USA; 2 Geisel School of Medicine at Dartmouth, Hanover, NH 03755 USA

**Keywords:** Primary care, Primary spine care, Spine-related disorders, Low back pain, Spine pain escalation of care, Academic primary care clinic, Chiropractic, Efficiency

## Abstract

**Background:**

Primary Spine Care (PSC) is an innovative model for the primary management of patients with spine-related disorders (SRDs), with a focus on the use of non-pharmacological therapies which now constitute the recommended first-line approach to back pain. PSC clinicians serve as the initial or early point of contact for spine patients and utilize evidence-based spine care pathways to improve outcomes and reduce escalation of care (EoC; e.g., spinal injections, diagnostic imaging, hospitalizations, referrals to a specialist). The present study examined 6-month outcomes to evaluate the efficiency of care for patients who received PSC as compared to conventional primary care. We hypothesized that patients seen by a PSC clinician would have lower rates of EoC compared to patients who received usual care by a primary care (PC) clinician.

**Methods:**

This was a retrospective observational study. We evaluated 6-month outcomes for two groups seen and treated for an SRD between February 01, 2017 and January 31, 2020. Patient groups were comprised of N = 1363 PSC patients (Group A) and N = 1329 PC patients (Group B). We conducted Pearson chi-square and logistic regression (adjusting for patient characteristics that were unbalanced between the two groups) to determine associations between the two groups and 6-month outcomes.

**Results:**

Within six months of an initial visit for an SRD, a statistically significantly smaller proportion of PSC patients utilized healthcare resources for spine care as compared to the PC patients. When adjusting for patient characteristics, those who received care from the PSC clinician were less likely within 6 months of an initial visit to be hospitalized (OR = .47, 95% CI .23–.97), fill a prescription for an opioid analgesic (OR = .43; 95% CI .29–.65), receive a spinal injection (OR = .56, 95% CI .33–.95), or have a visit with a specialist (OR = .48, 95% CI .35–.67) as compared to those who received usual primary care.

**Conclusions:**

Patients who received PSC in an academic primary care clinic experienced significantly less escalation of their spine care within 6 months of their initial visit. The PSC model may offer a more efficient approach to the primary care of spine problems for patients with SRDs, as compared to usual primary care.

**Supplementary Information:**

The online version contains supplementary material available at 10.1186/s12998-022-00411-x.

## Background

The management of spine-related disorders (SRDs), including back pain, is the largest contributing factor to increased outpatient healthcare utilization and expenditures [1]. Spending for low back and neck pain has become accountable for the highest costs in US health care, with an estimated spending of $134.5 billion [2], and increased expenditures for spine care interventions have not correlated with improved outcomes [3]. Moreover, there is insufficient evidence to justify the use of many invasive and expensive spine care procedures [4, 5]. The medical management of back pain can be also be hazardous, as back pain is the most common condition for which opioids are prescribed [6]. Therefore, it is critical to implement guideline-concordant clinical pathways that improve outcomes, improve the efficiency of care, and reduce escalation of care (EoC)—the unnecessary use of healthcare resources—for the management of patients with SRDs.

Most patients with back pain in the U.S. are initially seen by a primary care clinician. Because many of the non-pharmacological treatment approaches recommended in clinical practice guidelines [7] such as spinal manipulation, acupuncture, and massage therapy are not taught in allopathic medical schools, it is often a challenge for primary care physicians (PCP) to operationalize these guidelines within their clinical settings. One promising solution is to embed within the primary care environment a dedicated spine care clinician who has the requisite knowledge and skills needed to manage patients with SRDs and provide guidance through the maze of spine treatment options [8]. This approach, known as Primary Spine Care (PSC) is an innovative model of for the management of SRDs. PSC is defined as management, case coordination, and follow-up of spine patients within a conventional clinical care setting, under the direction of a dedicated PSC clinician [8–10]. The PSC clinician practices without need of referral as a primary contact clinician (ideally, a portal of entry clinician)who provides non-pharmacological care and coordinates the primary spine care of patients with spine problems from presentation through discharge. Thus, the PSC clinician can serve as the initial or early poin*t* of contact for spine patients, and as an alternative to the usual primary care pathway, which often requires referral for non-pharmacological care and is therefore less efficient from the start [11].

### Objective

Evidence-based non-pharmacological management of SRDs has been reported to be associated with less healthcare utilization and lower costs as compared to usual medical care [12–14]. Recently, implementation of PSC in a conventional primary care setting was associated with a trend toward lower expenditures for spine care [15], leading the authors to hypothesize that implementation of PSC may result in less escalation of care (e.g., spinal injections, hospitalizations, diagnostic imaging, referrals to a specialist), and thus improve efficiency. Efficiency in healthcare can be defined as a comparison of delivery system outputs such as doctor visits and health outcomes with inputs such as cost, time, and resources [16]. The objective of the present study was to compare PSC versus usual care with regard to the efficiency of spine care in an academic primary care setting.

## Methods

### Overall approach

Following up on a previous report of initial outcomes [15], the aim of this study was to evaluate outcomes for PSC vs. conventional primary care (PC) after three years following implementation of the PSC model. Employing a retrospective observational design, we analyzed electronic health records for patients seen for a primary diagnosis of a spine-related disorder at an academic primary care facility. For all patients, we measured 6-month outcomes, with a focus on encounters indicative of the escalation of spine care. We hypothesized that among patients with SRDs, patients who received PSC care would showcase lower rates of EoC, including hospitalizations, ED visits, spinal injections, visits to a specialist, and prescription fills for opioid analgesics as compared to those who received PC.

### Primary spine care (PSC): implementation and barriers

PSC services were provided by a Doctor of Chiropractic with a MS degree in sport science and rehabilitation and 5 years of experience in clinical practice. He completed a residency in chiropractic at a Veterans Health Administration hospital, followed by a university-based clinical fellowship in PSC, and certification in PSC by the University of Pittsburgh. The PSC clinician was embedded within the flagship primary care facility of an academic medical center practicing within a multi-clinician setting. The embedding of the PSC clinician was met with approval by the primary care clinicians and support staff. Patients presenting with LBP were predominantly seen by a primary care clinician and received standard care for LBP with most patients additionally being referred by the primary care clinician to the PSC clinician. Embedding the PSC clinician in the primary care team facilitated real-time, two-way communication in managing LBP as the PSC clinician could easily share findings, diagnosis, and treatment plan with the referring clinician, in addition to internal communication via the electronic medical record [17].

Additionally, implementing the PSC model within this setting did include several barriers. The first barrier was *explicit bias*, which is the belief that providers other than medical physicians are ill-suited or untrained to assume such a role in primary care. A second barrier was s*tructural bias—*limited insurance reimbursement for non-medical providers. The last barrier was *implicit bias*, which was associated with physician and administrator lack of familiarity with the PSC clinician’s training, expertise, and competencies [17].

However, these barriers were successfully addressed. For example, e*xplicit bias* was successfully addressed by communicating the evidence supporting the suitability of non-physicians in treating pain from spine disorders. *Structural bias* was overcome because the availability of the PSC clinician effectively reduced “leakage” of patients to external providers, and the institution realized cost savings in the care of the self-insured employee population. *Implicit bias* was pre-emptively tackled through one-on-one education of physicians and administrators using evidence-based literature [17].

PSC clinicians are experts in the diagnosis of SRDs, and in a range of conservative non-pharmacological therapies that constitute first-line, guideline-concordant treatment options for spinal pain. The PSC clinician uses evidence-based spine care pathways for clinical decision support that typically include stratification and management according to patient symptoms, developing a working diagnosis, and addressing biopsychosocial factors [11]. The guidelines and pathway utilized by the DC included the Clinical Reasoning in Spine Pain® (the CRISP® protocols) [18, 19] systematic approach. In this pathway, practitioners can maximize benefits to patients and practice—these protocols were developed based on the vast literature on the mechanisms, etiology, diagnosis and management of patients with spine related disorders (SRDs) [18, 19]. The approach provides a framework for the practitioner to apply existing evidence, knowledge and techniques to establish a diagnosis and management strategy for each patient [8].

### Participants and group assembly

This study employed a retrospective cohort design, which allows for a longitudinal evaluation of 180-day outcomes after each subject’s first clinical encounter (index date). Study subjects included all adult patients 18 years and older who presented with a new primary diagnosis of a spine-related disorder (SRD). A listing of codes pertaining to SRDs utilized for this study is provided in the Additional file 1: Table S2. Patients with a visit to the same clinician for primary diagnosis of an SRD within the time period of 1–30 days prior to the index date were excluded, and those with any visit for a primary diagnosis of SRD within the time period of 31–120 days prior to the index date were also excluded. The purpose of these exclusionary periods was to capture subjects with a new rather than ongoing complaint of SRD, but allow for referrals to the PSC clinician. Additionally, the following exposure variables were utilized in extracting the data: primary diagnosis, age of patient at index, sex of patient, patient race, marital status, education level, and patient employment status.

Thus, as a result of the inclusion/exclusion criteria outlined in the data extraction code (using the criteria outlined above), all patients who fit the study parameters and criteria were included in the analyses. In this process of data extraction, two groups were assembled for analysis: Group A—all patients seen by the PSC clinician on or following the index date of 02/01/2017; and Group B (comparison group) a sample of patients seen by a primary care clinician at the same facility for a new primary diagnosis of an SRD on or following 02/01/2017 and not seen by the PSC clinician. Thus, Group A received primary spine care, and Group B received conventional primary care. One DC provided the primary spine care, while 79 primary care clinicians provided primary care within this academic medical center. In this context, conventional primary care typically consisted of self-care advice, prescription for medication and/or referral to another clinician, most often a physical therapist. Usual primary care was provided by Family Medicine or Internal Medicine physicians, physician assistants, or nurse practitioners.

### Study design

**Fig. 1:**
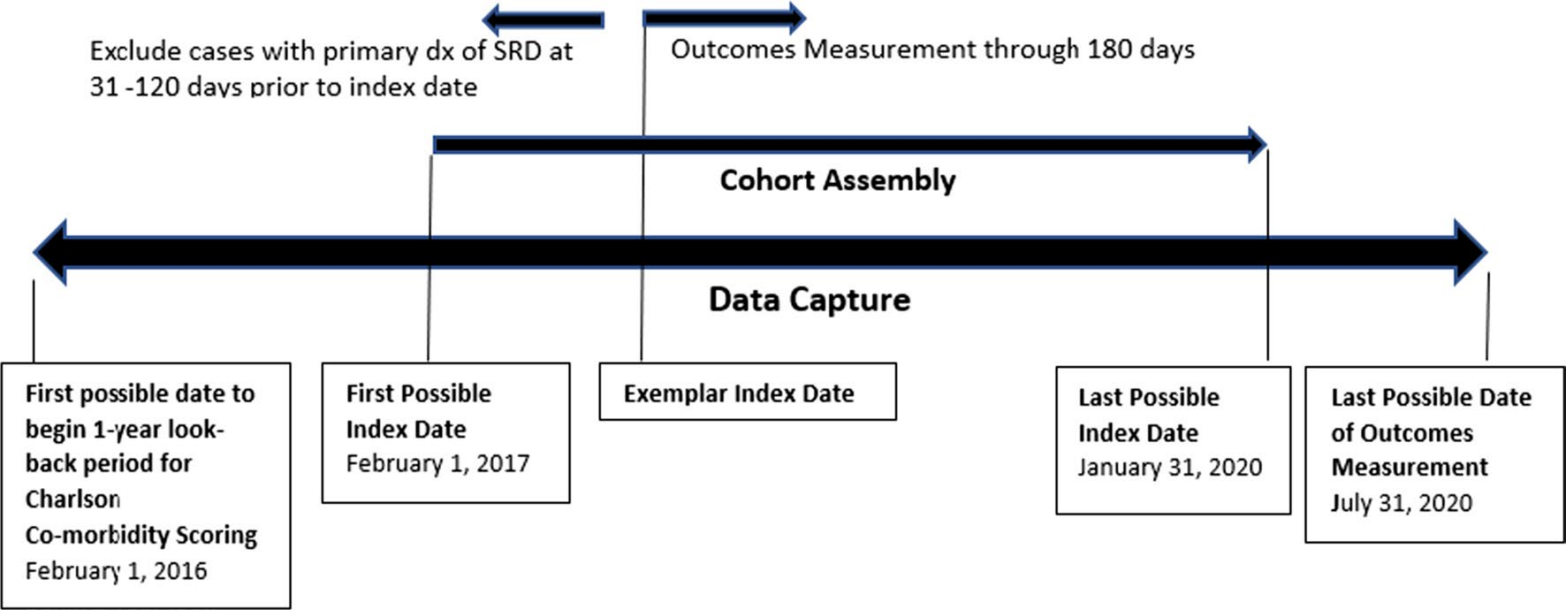
Data capture and cohort assembly

We collected data recorded in patient electronic health records. Data collection spanned a 12-month period preceding introduction of the PSC model on 02/01/17, and the 42-month period following that date (Fig. 1). Patient demographic data from the 1-year period preceding the date of introduction of the PSC model (02/01/2017) included calculated Charlson Comorbidity Index scores. We measured clinical outcomes for up to 180 days following the index date. All aspects of the current study were approved by our Institution’s Review Board (IRB).

### Statistical analysis

We conducted descriptive statistics on demographic data (e.g., frequencies for age categories, gender, race/ethnicity, marital status, employment status, Charlson comorbidity score, and primary diagnosis). Additionally, we conducted both descriptive statistics and Pearson chi-square analyses (using Fisher’s Exact test for cells counts < 5) to examine rates and percentages for outcomes between the two groups. The outcomes examined were the frequency of escalated care encounters associated with a primary diagnosis of SRD, including emergency department (ED) visits, diagnostic imaging, spinal injections, hospitalizations, surgeries, and referrals to a specialist, as well as prescription fills for opioid analgesics. We also conducted *t*-tests to assess for mean differences in patient age and Charlson Comorbidity score between the two groups. Response options for Marital Status and Employment Status were combined to create fewer categories for ease of analysis. Moreover, we calculated the Number Needed to Treat (NNT) based on the rates of outcomes in each group/cohort for each outcome presented in the study [20, 21].

Additionally, to account for selection bias, we conducted a series of regression models to evaluate outcomes while adjusting for patient characteristics (i.e., covariates). We initially conducted inverse probability of treatment models (i.e., inverse weighted propensity score models) to account for bias; however, we could not achieve an optimal balance in the covariates on the exposure variable (i.e., between the two groups). Thus for the present analyses, we present regression models adjusted for the following covariates that were significantly different between the two groups (see Table 1): age, gender, employment status, Charlson score, and primary diagnosis at index. Specifically, we conducted binary logistic Generalized Linear Regression Models (GLM) to model each clinical outcome while controlling for patient characteristics. Prior data simulation studies have indicated that regression models adjusting for covariates can adequately detect treatment effects [22]. All outcomes were dichotomous (coded as ‘yes/no’ within 6 months of initial visit, if the outcome was present) for analyses and odds ratios (OR) are reported in the results. All descriptive and regression analyses were conducted using IBM SPSS (Version 23). Additionally, this manuscript was prepared in accordance to STROBE guidelines for cohort studies [23].

**Table 1:** Patient characteristics for groups A (PSC patients) and B (PC patients) (*N* = 2692)

Characteristics	Group A (PSC Patients) n = 1363	Group B (PC Patients) n = 1329	Chi-square/t-test	*p*-value
Age (mean)			*t* = − 10.45	*p* < .001
Age in years at index (initial visit)	48	54.5*		
Sex (%)			10.06	*p* = .002
Female	64%*	58%		
Race/ethnicity (%)			4.07	*p* = .91
White/Caucasian	95%	96%		
Marital status (%) (n = 2687)				
Cohabiting	57.5%	58.3%	0.199	*p* = *.655*
Not cohabiting	15.1%	15.1%		
Employment status (%)			55.15	p < .001
Employed (FT, PT, self-employed)	66.7%	54.1%		
Retired	16.2%	26.9%		
Unemployed	10.5%	12.0%		
Other (student, unknown)	6.6%	6.9%		
Primary diagnosis (pain source) at index date—pain source (%)			1263.12	*p* < .001
Radicular	7.7%	17.8%		
Disc	18.3%	2.6%		
Facet or segmental dysfunction	55.8%	4.5%		
Myofascial	3.2%	12.6%		
Non-specific back pain	14.2%	56.7%		
Other	0.9%	5.8%		
Charlson Comorbidity Score (mean)	0.63	0.95*	*t* = − 5.46	*p* < .001

Frequencies presented for *most common categories*. Categories for marital status and employment status were collapsed for ease of analysis. p-values are from Pearson chi-square analyses or t-tests. Mean Charlson score and age based on a t-test. Race/Ethnicity reports Fisher’s Exact probability. Mean age was significantly higher for patients in Group B (PC patient) (p < .001). Frequency of females was significantly higher in Group A (p = .002), and mean Charlson score was significantly higher in Group B (p < .001)

## Results

### Descriptive statistics

Data from 2692 patients were included in the present analysis—and the following two groups of patients were assembled and examined: Group A (1363 PSC patients) and Group B (1329 PC patients). Table 1 displays patient characteristics for both patient groups. The two patient groups were comparable in marital status and race/ethnicity (where over 90% of the two samples identified as White/Caucasian). However there were differences between the two patient groups: mean age was significantly higher for patients in Group B (PC patients) (p < 0.001) compared to Group A (PSC patients). Additionally, the frequency of females was significantly higher in Group A compared to Group B (p = 0.002), and mean Charlson score was significantly higher in Group B (p < 0.001) (Table 1).

### Outcomes

**Fig. 2:**
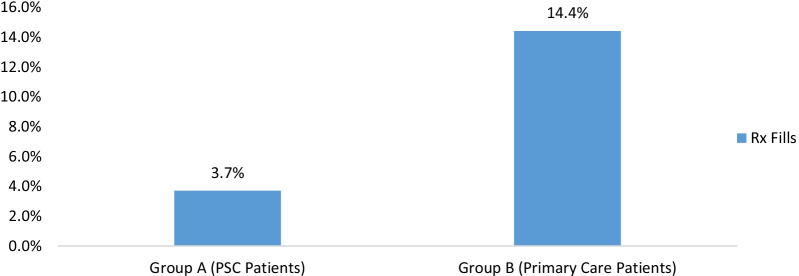
Percentage of patients who filled a prescription for opioid analgesic within six months of initial visit. *Note*: Percent of patients who filled a prescription (Rx) was significantly higher in Group B χ^2^ = 93.9, p < .001 (Fischer’s exact). This graph presents the frequency of patients (based on a dichotomized yes/no variable) who filled a prescription within 6 months of Index

The frequency of patients who filled a prescription for opioid pain medication 6 months after an initial visit was significantly higher in Group B χ^2^ = 93.9, p < 0.001 (Fischer’s Exact) compared to Group A (PSC patients) (Fig. 2).

**Fig. 3:**
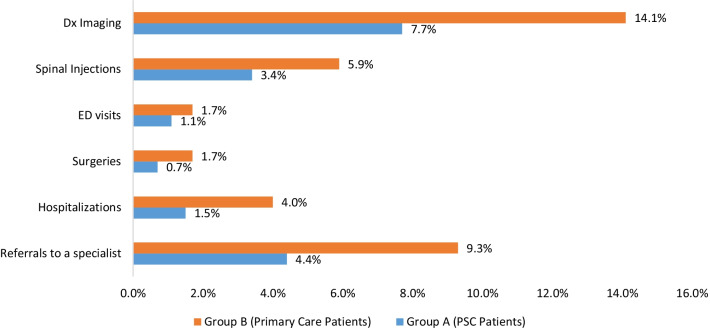
Frequency of Escalated Spine Care Encounters in the PSC and PC Groups (N = 2692). *Note*: Percentages are *frequencies of patients* in each group that utilized the various healthcare resources; Differences in the frequencies between the groups was determined using Pearson chi-square test statistics. All comparisons were significant at *p* < .05 except for Emergency Department (ED) visits. Dx = Diagnostic imaging

Additionally, within six months of an initial visit for an SRD, a significantly smaller proportion of PSC patients utilized healthcare resources compared to PC patients (Group B). Specifically, a smaller percentage of PSC patients filled prescriptions for opioid analgesics (3.7% vs. 14.4%, p < 0.001), had hospitalizations (1.5% vs. 4%, p < 0.001), had surgeries (0.7% vs. 1.7%, p = 0.03), had referrals to a specialist (e.g., the facility’s spine center) (4.4% vs. 9.3%, p < 0.001), had spinal diagnostic imaging (7.7% vs. 14.1%, p < 0.001), and had spinal injections (3.4% vs. 5.9%, p = 0.002) (Fig. 3; Additional file 1: Table S1).

Moreover, the NNT for the various outcomes ranged between 16 (for diagnostic imaging) and approximately 160 (for ED visits). These results indicate that on average 16 individuals would need to receive treatment from a PSC in order for one additional patient not to experience the outcome of Diagnostic Imaging, for example (see Additional file 1: Table S1).

However, PSC patients had significantly higher rates of visits to the primary care clinic compared to PC patients. PSC patients had an average of 8.6 visits compared to an average of 4.5 visits for PC patients (this difference in means was significant *t* = 15.92; df = 2550 *p* < 0.001). For total general visits, PSC patients had an average of 3.8 compared to 2.2 for PC patients, which was a significant difference *t* = 17.79; df = 2370; *p* < 0.001.

When controlling for patient characteristics (age, gender, employment status, primary diagnosis, and Charlson comorbidity score), PSC patients were less likely to experience escalation of spine care compared to PC patients. Specifically, PSC patients were 53% less likely to be hospitalized OR = 0.47, 95% CI 0.23–0.97), 57% less likely to fill a prescription for an opioid analgesic OR = 0.43; 95% CI 0.29–0.65), 44% less likely to obtain spinal injections (OR = 0.56, 95% CI 0.33–0.95), and 52% less likely to have a visit with a specialist OR = 0.48, 95% CI 0.35–0.67) compared to PC patients (Table 2).

**Table 2:** Likelihood of escalation of care for patients who received primary spine care versus usual primary care

Outcome	OR	95% CI OR	*p*-value
Hospitalizations (n = 73)	.47	.23–.97	p = .04
Surgeries (n = 32)	.51	.19–1.36	p = .18
Emergency department (ED) visits (n = 38)	.91	.37–2.22	p = .83
Pain prescription fills (n = 243)	.43	.29–.65	p < .001
Diagnostic imaging of the spine (n = 292)	.87	.63–1.21	p = .41
Spinal injections (n = 124)	.56	.33–.95	p = .03
Specialist visits (n = 184)	.48	.35–.67	p < .001

Group B (PC Patients) served as the referent (comparison) group. All outcomes coded as binary (0/1, “no/yes”) for binary logistic GLM models (controlling for covariates: age, gender, employment status, primary diagnosis, and Charlson score; although depending on the clinical outcome, not all covariates were significant in the various models). OR = odds ratio from the regression model (equivalent to the exp(b) statistic). P-values are from the Wald test reported for the exposure variable (group/cohort)

## Discussion

In this study, we evaluated an alternative approach (PSC model) to spine care, and report outcomes associated with implementation of the model within an academic primary care clinic. This is the first paper to compare 6-month outcomes for PSC within a conventional, academic primary care setting. The results demonstrated that within six months of an initial visit for an SRD, a significantly smaller proportion of PSC patients had escalation of spine care as compared to PC patients. Exceptions included ED visits and surgeries, where we did not find a significant difference between the two groups (based on Pearson chi-square analyses and/or the GLM regression models)—most likely because the outcomes were so rare. As compared to PC patients and with controlling for patient characteristics, PSC patients were less likely within 6 months of an initial visit to be hospitalized for spinal pain, fill a prescription for an opioid analgesic, receive a spinal injection, or visit a specialist for a complaint of SRD.

The present study’s findings are consistent with and expand upon a previous report, which demonstrated that implementation of the PSC model within a conventional, academic primary care setting was associated with a trend toward reduced total expenditures for spine care, and lower odds of diagnostic imaging of the spine, as compared with usual primary care [15]. Similarly, in a study of older Medicare beneficiaries who initiated long-term care for chronic low back pain, the rate of EoC encounters was significantly lower as compared to those who initiated care with spinal manipulative therapy [24]. Furthermore, a large cohort study conducted by Stevans et al. [25] found that the transition from acute to chronic LBP was substantial and early exposure to guideline non-concordant care was significantly associated with the transition to chronic LBP (after adjusting for patient and clinical characteristics). The authors concluded that an emphasis on implementing guideline concordant care within a primary care setting was integral to reducing the development of chronic LBP [25].

A recent survey also reported high rates of patient satisfaction with PSC treatments received in an academic primary care setting [26]. The results of this study are also consistent with a recent evaluation of a clinical model characterized by a patient-centered approach and standardized, best-practice clinical protocols, similar to the PSC model, which demonstrated lower costs when compared to non-standardized approaches to chiropractic care [27]; no cost comparison was made with conventional primary care, however.

The PSC model appears to be a valuable innovation in primary care because it supports evidence-based practice and may improve efficiency through reduced EoC, thereby improving the quality of care while reducing costs. Similar to imbedding mental health clinicians within a primary care clinic, the PSC model is collaborative and not just co-located, parallel care. PSC promises to reduce the burden primary care clinicians often experience in caring for patients with back pain, theoretically increasing patient access to primary care, and at the same time improving the efficiency and value of spine care. The PSC model may hypothetically be implemented in a private practice setting, not imbedded within primary care, given the support of payors and policy makers, and strong collaboration between PC clinicians and specialists. However, this would require further study.

### Limitations

The present findings should be taken with certain limitations in mind. Spine care outside of the academic health center was not examined. Additionally, the available data contained few variables pertaining to other related patient outcomes that may influence clinical outcomes such as general psychological state (e.g., depressive disorders, anxiety, fear avoidance or exposure to stress), which prior studies have noted to be associated with SRDs [28]. In addition to the lack of psychological variables in the dataset, we also did not have clinical outcome measures such as those assessing patients’ level of disability or patient self-reports of pain ratings, or a Global Index of Change, which would have been useful for examining differences in these two groups of patients. Future studies should consider including these clinical outcome variables when examining differences in the care received between primary spine care and primary care patients. Although we adjusted for patient characteristics in the regression models, the comparisons may have been confounded by unmeasured factors. Additionally, the rates for the various primary diagnosis categories significantly differed between the two groups, which may have influenced the regression model results. For example, visits to a PC clinician may include several different complaints being addressed along with a complaint for spine pain, while spine pain is invariably the main complaint in a visit to the PSC. The findings of this observational study should be confirmed with a randomized control trial that accounts for unmeasured confounders and examines longer-term outcomes of effectiveness and costs. Due to the way the data were extracted for these analyses—utilizing code with specific inclusion and exclusion criteria, we did not have information to how many potential patients may have been lost to follow-up.

In this implementation of the PSC model, the majority of PSC patients saw a primary care clinician first; thus, the ideal scenario in which the PSC clinician acts as the first point of contact for spine patients has yet to be realized. Changes to long-established clinical practice patterns, however beneficial to both patient and clinician, are more likely to be evolutionary rather than revolutionary. Thus, patients with pathological pain requiring escalation of care were probably less likely to be referred to the PSC clinician. Nevertheless, in the clinic studies, clinician attitudes appear to favor full implementation: in an internal performance improvement survey, 88% of primary care physicians reported that PSC made it easier for them to care for patients with spine pain, and 100% accepted the PSC clinician as the first or initial contact for an SRD [17]. Moreover, generalizability of the present study’s findings is limited to patients treated for an SRD within this academic medical center and may not be generalizable to all back pain patients.

## Conclusions

In our evaluation of this innovative model of spine care, patients who were seen and treated by a PSC clinician embedded in an academic primary care clinic experienced significantly less escalation of their spine care within six months of their initial visit and filled significantly fewer prescriptions for opioid pain medication. The PSC model facilitates greater compliance with current evidence-based guidelines for the management of spine care and may offer a more efficient approach to the primary care of spine problems, as compared to conventional primary care.

## Supplementary Information

**Additional file 1.** Appendix.

## Data Availability

The study dataset is not available for sharing, according to the terms of a data use agreement with a third party.

## Abbreviations

95% CI: 95 Percent confidence interval
EoC: Escalation of care
GLM: Generalized linear regression models
PC: Primary care
PCP: Primary care physician
PSC: Primary spine care
NNT: Number needed to treat
SRD: Spine-related disorder
