# Supplementary material for: Efficiency of primary spine care as compared to conventional primary care: a retrospective observational study at an Academic Medical Center
Source: Chiropr Man Therap. 2022 Jan 6;30:1. doi: 10.1186/s12998-022-00411-x (PMC8740480; doi:10.1186/s12998-022-00411-x)
Supplement: Supplementary file 1 — Additional file 1. Appendix. [file 12998_2022_411_MOESM1_ESM.docx]

**Appendix**

Table A1. Comparison of Frequencies of Patients Utilizing Escalation of Spine Care in Groups A (PSC patients) and B (PC patients) and the Number Needed to Treat

|  | Group A  (PSC Patients)  N = 1,363 | Group B  (PC Patients)  N = 1,329 | *Χ*^2^ | p-value | NNT | 95% CI (NNT) |
| --- | --- | --- | --- | --- | --- | --- |
| Spinal Injections | 3.4% | 5.9% | 9.58 | p = .002 | 40 | 24.5 - 109.5 |
| Diagnostic Imaging | 7.7% | 14.1% | 28.36 | p <.001 | 16 | 11.5 - 24.8 |
| Referrals to a specialist | 4.4% | 9.3% | 25.67 | p <.001 | 20 | 14.6 - 32.9 |
| Emergency Department (ED) visits | 1.1% | 1.7% | 1.9 | p = .19 | 159 | 66.0 - 383.0 |
| Surgeries | 0.7% | 1.7% | 4.9 | p = .03 | 100 | 54.7 - 601.0 |
| Hospitalizations | 1.5% | 4% | 16.21 | p <.001 | 40 | 26.7 - 77.1 |

*Note.* Percentages are frequencies of patients in each group that utilized the various healthcare resources; *Χ*^2^  = Pearson chi-square test statistics. NNT = Number Needed to Treat

Table A2. Listing of Codes for SRD based on ICD-9 and ICD-10

| **ICD-9 Description** | **ICD-9 Code** | **ICD-10 Description** | **ICD-10 Code** |
| --- | --- | --- | --- |
| Instability | 718.88 | Other specific joint derangements of unspecified joint, not elsewhere classified | M24.80 |
| Inflammatory/Ankylosing spondylosis | 720 | Ankylosing spondylitis of unspecified sites in spine | M45.9 |
| Spondylosis | 721 | Spondylosis without myelopathy or radiculopathy, cervical region | M47.812 |
| Spondylosis w/myelopathy | 721.1 | Other spondylosis with myelopathy, cervical region | M47.12 |
| Spondylosis | 721.2 | Spondylosis without myelopathy or radiculopathy, thoracic region | M47.814 |
| -- | -- | Spondylosis without myelopathy or radiculopathy, lumbar region | M47.816 |
| Spondylosis | 721.3 | Spondylosis without myelopathy or radiculopathy, lumbosacral region | M47.817 |
| Spondylosis w/myelopathy | 721.41 | Other spondylosis with myelopathy, thoracic region | M47.14 |
| Spondylosis w/myelopathy | 721.42 | Other spondylosis with myelopathy, lumbar region | M47.16 |
| DISH | 721.8 | Spondylopathy, unspecified | M48.9 |
| Disc herniation | 722 | Other cervical disc displacement, unspecified cervical region | M50.20 |
| -- | -- | Cervical disc disorder with radiculopathy | M50.10 |
| -- | -- | Lumber disc herniation | M51.16 |
| Disc herniation | 722.1 | Other intervertebral disc displacement, lumbar region | M51.26 |
| Disc herniation | 722.1 | Other intervertebral disc displacement, lumbosacral region | M51.27 |
| Disc herniation | 722.11 | Other intervertebral disc displacement, thoracic region | M51.24 |
| Disc herniation | 722.11 | Other intervertebral disc displacement, thoracolumbar region | M51.25 |
| Disc degeneration | 722.4 | Other cervical disc degeneration, unspecified cervical region | M50.30 |
| Disc degeneration | 722.51 | Other intervertebral disc degeneration, thoracic region | M51.34 |
| Disc degeneration | 722.51 | Other intervertebral disc degeneration, thoracolumbar region | M51.35 |
| Disc degeneration | 722.52 | Other intervertebral disc degeneration, lumbar region | M51.36 |
| Disc degeneration | 722.52 | Other intervertebral disc degeneration, lumbosacral region | M51.37 |
| Disc herniation w/myelopathy | 722.71 | Cervical disc disorder with, myelopathy, unspecified cervical region | M50.00 |
| Disc herniation w/myelopathy | 722.72 | Intervertebral disc disorders with myelopathy, thoracic region | M51.04 |
| Disc herniation w/myelopathy | 722.72 | Intervertebral disc disorders with myelopathy, thoracolumbar region | M51.05 |
| Disc herniation w/myelopathy | 722.73 | Intervertebral disc disorders with myelopathy, lumbar region | M51.06 |
| Disc herniation w/myelopathy | 722.73 | Intervertebral disc disorders with myelopathy, lumbosacral region | M51.07 |
| Post laminectomy syndrome | 722.81 | Postlaminectomy syndrome, not elsewhere classified | M96.1 |
| Post laminectomy syndrome | 722.83 | Postlaminectomy syndrome, not elsewhere classified | M96.1 |
| Post laminectomy syndrome | 722.83 | Postlaminectomy syndrome, not elsewhere classified | M96.1 |
| Disc space infection | 722.91 | Other cervical disc disorders, unspecified cervical region | M50.80 |
| Disc space infection | 722.91 | Cervical disc disorder, unspecified, unspecified cervical region | M50.90 |
| Disc space infection | 722.92 | Discitis, unspecified, thoracolumbar region | M46.45 |
| Disc space infection | 722.92 | Other intervertebral disc disorders, thoracic region | M51.84 |
| Disc space infection | 722.92 | Other intervertebral disc disorders, thoracolumbar region | M51.85 |
| Disc space infection | 722.93 | Other intervertebral disc disorders, lumbar region | M51.86 |
| Disc space infection | 722.93 | Other intervertebral disc disorders, lumbar region | M51.87 |
| Spinal stenosis | 723 | Spinal stenosis, cervical region | M48.02 |
| Pain, Neck | 723.1 | Cervicalgia | M54.2 |
| Radiculitis | 723.4 | Radiculopathy, cervical region | M54.12 |
| Radiculitis | 723.4 | Radiculopathy, cervicothoracic region | M54.13 |
| Spinal stenosis | 724.01 | Spinal stenosis, thoracic region | M48.04 |
| Spinal stenosis | 724.02 | Spinal stenosis, lumbar region | M48.06 |
| Pain Thoracic Spine | 724.1 | Pain in thoracic spine | M54.6 |
| Pain lower back | 724.2 | Low back pain | M54.5 |
| Sciatica | 724.3 | Sciatica, unspecified site | M54.30 |
| Pain, Radicular | 724.4 | Radiculopathy, thoracic region | M54.14 |
| Pain, Radicular | 724.4 | Radiculopathy, thoracolumbar region | M54.15 |
| Radiculitis | 724.4 | Radiculopathy, thoracic region | M54.14 |
| Pain, Radicular | 724.4 | Radiculopathy, lumbar region | M54.16 |
| Pain, Radicular | 724.4 | Radiculopathy, lumbosacral region | M54.17 |
| Radiculitis | 724.4 | Radiculopathy, thoracolumbar region | M54.15 |
| Radiculitis | 724.4 | Radiculopathy, lumbar region | M54.16 |
| Radiculitis | 724.4 | Radiculopathy, lumbosacral region | M54.17 |
| Coccyxgodynia | 724.79 | Sacrococcygeal disorders, not elsewhere classified | M53.3 |
| Costochondritis | 733.6 | Chondrocostal junction syndrome [Tietze] | M94.0 |
| Kyphosis | 737.1 | Postural kyphosis, site unspecified | M40.00 |
| Kyphosis | 737.1 | Unspecified kyphosis, site unspecified | M40.209 |
| Scoliosis w/o neurogenic cause | 737.3 | Other idiopathic scoliosis, site unspecified | M41.20 |
| Spondylolisthesis | 738.4 | Spondylolisthesis, site unspecified | M43.10 |
| Spondylolisthesis congenital | 756.12 | Congenital spondylolisthesis | Q76.2 |
| -- | -- | Segmental and somatic dysfunction of cervical region | M99.01 |
| -- | -- | Segmental and somatic dysfunction of thoracic region | M99.02 |
| -- | -- | Segmental and somatic dysfunction of lumbar region | M99.03 |
| -- | -- | Segmental and somatic dysfunction of sacral region | M99.04 |
| -- | -- | Segmental and somatic dysfunction of pelvic region | M99.05 |
| -- | -- | Segmental and somatic dysfunction of rib cage | M99.08 |
| -- | -- | Myofascial pain | M79.18 |
| -- | -- | Myalgia | M79.1 |
| Sprain/whiplash | 847 | Sprain of ligaments of cervical spine, initial encounter | S13.400A –  S13.499A |
| Sprain | 847.1 | Sprain of joints and ligaments of other parts of neck, initial encounter | S13.800A –  S13.899A |
| Sprain | 847.1 | Sprain of ligaments of thoracic spine, initial encounter | S23.300A –  S23.399A |
| Sprain | 847.1 | Sprain of other unspecified parts of thorax, initial encounter | S23.800A-  S23.899A |
| Sprain | 847.1 | Sprain of ligaments of lumbar spine, initial encounter | S33.500A-  S33.599A |
| Strain, non traumatic musculoskeletal | 847.3 | Sprain of other parts of lumbar spine and pelvis, initial encounter | S33.800A-  S33.899A |
